# Osteogenic and Chondrogenic Potential of Periosteum-Derived Mesenchymal Stromal Cells: Do They Hold the Key to the Future?

**DOI:** 10.3390/ph14111133

**Published:** 2021-11-08

**Authors:** Madhan Jeyaraman, Sathish Muthu, Prakash Gangadaran, Rajni Ranjan, Naveen Jeyaraman, Gollahalli Shivashankar Prajwal, Prabhu Chandra Mishra, Ramya Lakshmi Rajendran, Byeong-Cheol Ahn

**Affiliations:** 1 Department of Orthopaedics, School of Medical Sciences and Research, Sharda University, Greater Noida 201306, Uttar Pradesh, India; madhanjeyaraman@gmail.com (M.J.); rajni.ranjan@sharda.ac.in (R.R.); 2 Department of Biotechnology, School of Engineering and Technology, Sharda University, Greater Noida 201310, Uttar Pradesh, India; 3 International Association of Stem Cell and Regenerative Medicine (IASRM), Greater Kailash, New Delhi 110048, Uttar Pradesh, India; info@iasrmglobal.org; 4 Department of Orthopaedics, Government Medical College and Hospital, Dindigul 624304, Tamil Nadu, India; 5 BK21 FOUR KNU Convergence Educational Program of Biomedical Sciences for Creative Future Talents, Department of Biomedical Sciences, School of Medicine, Kyungpook National University, Daegu 41944, Korea; prakashg@knu.ac.kr; 6 Department of Nuclear Medicine, School of Medicine, Kyungpook National University, Kyungpook National University Hospital, Daegu 41944, Korea; 7 Department of Orthopaedics, Atlas Hospitals, Tiruchirappalli 620002, Tamil Nadu, India; naveenjeyaraman@yahoo.com; 8 Department of Orthopaedics, Mallika Spine Centre, Guntur 522001, Andhra Pradesh, India; prajwalgs1894@gmail.com

**Keywords:** periosteum, mesenchymal stromal cells, chondrogenesis, osteogenesis

## Abstract

The periosteum, with its outer fibrous and inner cambium layer, lies in a dynamic environment with a niche of pluripotent stem cells for their reparative needs. The inner cambium layer is rich in mesenchymal progenitors, osteogenic progenitors, osteoblasts, and fibroblasts in a scant collagen matrix environment. Their role in union and remodeling of fracture is well known. However, the periosteum as a source of mesenchymal stem cells has not been explored in detail. Moreover, with the continuous expansion of techniques, newer insights have been acquired into the roles and regulation of these periosteal cells. From a therapeutic standpoint, the periosteum as a source of tissue engineering has gained much attraction. Apart from its role in bone repair, analysis of the bone-forming potential of periosteum-derived stem cells is lacking. Hence, this article elucidates the role of the periosteum as a potential source of mesenchymal stem cells along with their capacity for osteogenic and chondrogenic differentiation for therapeutic application in the future.

## 1. Introduction

**Figure 1:**
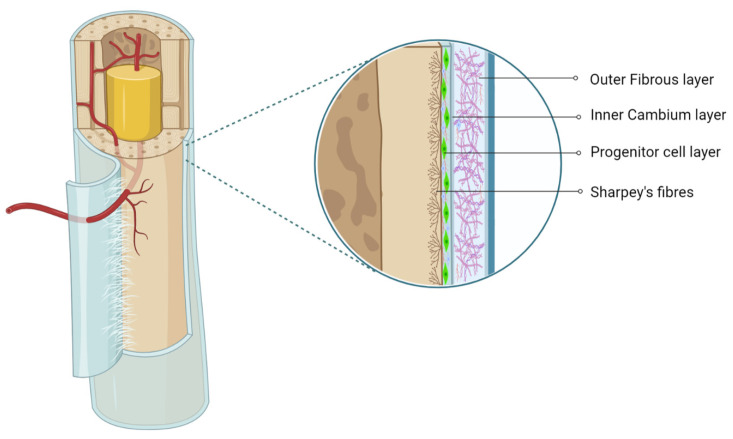
Anatomy of periosteum. Created with BioRender.com.

The periosteum is a thin fibrous layer, which forms an outer covering of the bone surface. It contains the outer fibrous layer and inner cambium layer, which possess significant osteogenic potential [1,2]. In 1867, Ollier claimed that the cambium layer is responsible for appositional bone growth [3]. The periosteum lies in a dynamic mechanically loaded environment and provides a niche for pluripotent stem cells [2,4]. The outer fibrous layer contains a superficial inelastic hypocellular layer with high content of the collagenous matrix, which contributes a rich vascular supply to the bone and skeletal muscle and provides a network of neural fibers, whereas the deep elastic hypocellular hypovascular layer features a high-collagen matrix, as shown in Figure 1 [5]. The inner cambium layer is hypercellular with mesenchymal progenitors, osteogenic progenitors, osteoblasts, and fibroblasts in fewer quantities in the collagen matrix [6]. The cambium layer is rich in vascularity and neural networks. The presence of pericytes in the cambium layer confers the periosteum with more osteoblastic potential.^1^ The regenerative potential of the periosteum differs with age and bony location [6].

## 2. Periosteum as MSC Source

The cambium layer of the periosteum contains mesenchymal progenitor cells which can be extrapolated as periosteum-derived MSCs (P-MSCs) [7,8,9]. Various studies have stated that the periosteum is the best cellular therapeutic agent for bone regeneration due to the multipotent nature at the single-cell level, higher proliferation and differentiation rate, and retention of differentiation ability in vitro and in vivo [10,11]. P-MSCs from load-bearing sites possess more osteogenic potential than flat bones [12]. In cases of fractures, quiescent P-MSCs induce chondrogenesis and osteogenesis, which helps in long-term integration with native bone [13,14].

Molecular analysis revealed the periostin gene, which is responsible for enhanced tissue response to injury of periosteum [15]. Duchamp de Lageneste et al. demonstrated the higher regenerative potential of periosteal cells than bone marrow-derived MSCs (BM-MSCs). They observed that P-MSCs integrate into callus and cartilage by day 10 [16]. Lineage analysis of P-MSCs demonstrated that P-MSCs are derived from the Prx-1 mesenchymal lineage, which contributes to cartilage and bone within the callus [16].

## 3. Characterization and Isolation of P-MSCs

P-MSCs exhibit multilineage differentiation potential both in vitro and in vivo. P-MSCs exert the regenerative potential by possessing the effects of paracrine signaling, direct cell–cell interactions, and extracellular vehicles [17,18,19]. They possess surface antigens such as MSC markers (CD-73, -90, and -105, MSCA-1, CD-9 and -13, STRO-1, SSEA-4, ScaI, SOX-2, Oct-4, and Nanog) [20,21,22], integrin markers (CD-29 and -49e) [23,24], adhesion molecules (CD-31, -44, -166, -54, and -146) [25,26], and MHC class markers (HLA-ABC) [27], whereas they lack HSC markers (CD-14, -33, -34, 45, and -133, and HLA-DR) [28].

Frey et al. demonstrated the immunohistochemical and molecular characterization of P-MSCs in periosteal tissue samples [29]. They identified the presence of osteoblasts (ALP, M-CSF, Cbfa-1/Runx2, RANK-L, and Osterix), osteoclasts (TRAP, CTR, and cathepsin K), chondrocytes (SOX-9), and dendritic cells (MHC-II) in the periosteum. Yang et al. identified discoidal domain receptor-2 (DDR2), a novel marker for isolating osteoblasts and osteoblast progenitors of the periosteum [30]. Deveza et al. identified positive markers (*Mx1*^Cre^, *ROSA*^Tomato^, and *Osteocalcin*^GFP^) and negative markers (*Mx1+Ocn−*) of periosteal progenitor cells in mice [31].

Mx1 and α-smooth muscle actin are potent markers of quiescent skeletal stem cells in adult periosteal tissues [32]. Gao et al. evaluated the potentialities of periosteal progenitor cells and BM-MSCs using FACS Nestin^+^ PDGFR-α^+^ CD45^−^ Ter119^−^ CD31^−^ LepR^+^ markers. They demonstrated that both Nestin^+^ PDGFR-α^+^ and LepR^+^ periosteal progenitor cells formed more colony-forming unit fibroblasts (CFU-Fs) than BM-MSCs [33]. Craniofacial-derived periosteal progenitor cells show a similar growth curve to tibial-derived periosteal progenitor cells. Craniofacial human periosteum-derived cells (hPDCs) are positive for skeletal stem and progenitor cell markers CD73, CD164, and podoplanin and negative for CD146, HSC, and endothelial lineage markers [34].

Olbrich et al. obtained human periosteal samples from the maxilla and mandible bones [35]. Those samples were subjected to type 9 collagenase for 90 min and were plated onto 75 cm^2^ culture flasks. Periosteal tissues were cultured in a DMEM/F-12 medium containing antimicrobials. The proliferated periosteal tissues from the fifth to seventh passages were used for further experiments. Hence, magnetic separation (MACS) is a more suitable separation method to isolate osteoprogenitors from the entire jaw periosteal cell population.

From human donors, a 1 cm^2^ proximal medial tibial segment was removed, and the periosteum was harvested. Periosteal tissues were plated onto the serum-free α-MEM medium. Further tissue digestion was performed using collagenase D enzyme for 1 h and resuspended in α-MEM medium containing antimicrobial solutions. Cells were allowed to grow to 80% confluence, collected from the second and third passages for a lineage differentiation assay, and evaluated for stem-cell surface markers. De Bari et al. stated that, regardless of donor age, the adult human periosteum contains cells that, upon enzymatic release and culture expansion, are multipotent MSCs at the single-cell level [36].

## 4. Intracellular Signaling in Osteogenic Differentiation

Osteogenesis plays a major role in bone formation and turnover, fracture healing, and osseointegration of implants. Osteogenesis is an interplay among osteoprogenitor cells, osteoblasts, osteocytes, and osteoclasts. Osteogenic differentiation is regulated by various signaling pathways governed by osteoprogenitor markers (ALP, collagen type-1 and -2, decorin, Runx-2/CBFA-1, osterix, CD-146/M-CAM, and matrix extracellular phosphoglycoprotein (MEPE)), osteoblast markers (ALP, BAP-1 and -31, collagen type 1, fibronectin, osteocalcin, osterix, and SPARC), and osteocyte markers (biglycan, DMP-1, FGF-23, fibronectin, MEPE, podoplanin, sclerostin, and SPARC).

Temporary hypoxia exposure to MSCs leads to downregulation of type 1 collagen and Runx-2, and upregulation of OPN, which limits the in vivo osteogenic potential of MSCs [37]. More than 48 h of exposure to hypoxia in vitro exhibited MSC apoptosis when transplanted in vivo. Hence, the duration of in vitro hypoxia plays a major role in osteogenesis and osteoblastic differentiation when transplanted in vivo. Hypoxia-induced MSC-bound osteogenesis is primarily due to the presence of hypoxia-inducible factor 1α (HIF1α) with downregulation of Cbf-α1 [38]. Hypoxia upregulates phosphorylation of STAT-3 and expression of VEGF in MSCs. Downregulation of STAT-3 signaling results in dysfunctional osteogenesis in vitro [39].

Cheng et al. demonstrated osteogenic enhancement and adipogenic suppression of mesenchymal progenitors by Msx-2 [40]. KIAA1199 is a cell migration-inducing protein (CMIP), which helps in migration and mobilization of osteoprogenitor cells in vivo. KIAA1199 interacts with the wingless type (Wnt) signaling pathway, which induces osteogenesis and bone remodeling [41]. Wu et al. observed that overexpression of IGF-binding protein 4 (IGFBP-4) inhibits osteogenic differentiation in rat MSCs [42]. Upregulation of retinoic acid signaling inhibits the activity of ALP, leads to mineralization of dental pulp MSCs, and decreases the expression of factors inducing osteogenesis [43]. Extracellular matrix proteins possess varying affinity (fibronectin > type 1 and 4 collagen ≥ vitronectin > laminin-1) for the osteogenic differentiation of MSCs [44].

Suehiro et al. found that zinc finger and homeobox-3 (ZHX-3) helps in the switching of undifferentiated MSCs to in vitro osteogenic differentiation [45]. A forkhead transcription factor (FOXO) provides bidirectional regulation of MSC differentiation toward osteogenic cells, with positive regulators being Runx-2, ALP, PCN, and ATF-4 and negative regulators being β-catenin and OCN [46]. Differential expression of miRNAs is implicated in proliferation, differentiation, oncogenesis, and stemness of MSCs. The miR-31 exosome is the key regulator of osterix, a bone-specific transcription factor, which modulates osteogenesis and osteobiology [47].

Homeobox genes are well documented in the literature for intracellular signaling for osteogenesis. Homeobox C10 (HOX-C10) downregulates in vitro osteogenic differentiation [22,48], whereas distal less homeobox (DLX)-2 [49] and -5 [50] and HOX-B7 [51] upregulate osteogenic differentiation. The trimethylation of lysine 4 of histone H3 (H3K4Me3) correlates with osteogenic differentiation [52]. WD repeat-containing protein 63 (WDR63) enhances ALP activity, mineralization of osteoid, and the expression of BSP, OSX, and RUNX2 in vitro, as well as osteogenesis in nude mice [52].

Long noncoding RNAs (lncRNAs), considered “transcriptional noise”, emerged as key genomic regulators in cellular and tissue engineering. lncRNAs regulate osteogenic differentiation by binding to transcription factors and chromatin modification. The various mechanisms of osteogenesis by lncRNAs are as follows: (a) maternally expressed gene-3 (MEG-3) dissociates SOX-2 and promotes MSC-bound osteogenesis by enhancing BMP-4 expression, (b) phosphorylation of IκBα and activation of NF-κB inhibits osteogenesis, (c) downregulation of HOXA-AS3 enhances osteogenesis by targeting EZH2, and (d) upregulation of HIF1α-AS1 and AK141205 promotes MSC-bound osteogenesis and osteoblastic differentiation, respectively [53].

Extracellular vehicles (containing miRNAs) have the potential for osteogenic differentiation. miR-675 promotes osteogenesis by inhibiting the recruitment of histone deacetylases to Runx-2-bound DNA sequences. miR-675 targets H19, which further downregulates the β-catenin pathway and results in downregulation of osteogenesis. Upregulation of HOTAIR (inhibits miR-17-5p) [54] and MEG-3 (inhibits miR-133a-3p) [55] inhibits osteogenic differentiation by downregulating Runx-2, ALP, and COL1A1.

To enhance cellular therapeutic options in regenerative medicine, electromagnetic fields act as an auxiliary modality in tissue regeneration. Kang et al. observed both positive and negative osteogenic differentiation of AD-MSCs when applied using appropriate (30/45 Hz at 1mT) electromagnetic fields [56]. This hypothesis could form a basis for the acceleration of tissue expansion in vitro on 3D scaffolds when exposed to an electromagnetic field [56]. Enhanced osteogenic differentiation was observed in BM-MSCs when exposed to modified titanium surface (hydrophilic sandblasted and acid-etched) [57].

## 5. Osteogenicity of P-MSCs

P-MSCs sorted with CD-90^+^ demonstrate higher osteogenic potential than unsorted P-MSCs whether in vitro or in vivo [58]. Hence, CD-90^+^ sorted P-MSCs represent the ideal cell source with higher osteogenic potential for bone regeneration. Upregulation of fibroblast growth factor (FGF)-2, -5, and -6 leads to early callus formation and maintains periosteal osteogenesis [59]. Upregulation of FGFR-1 and -2 was observed in proliferating periosteal mesenchyme [59]. Upregulated expression of Jag-1 and Notch-2 genes was observed in MSCs of the healing periosteum [60].

**Figure 2:**
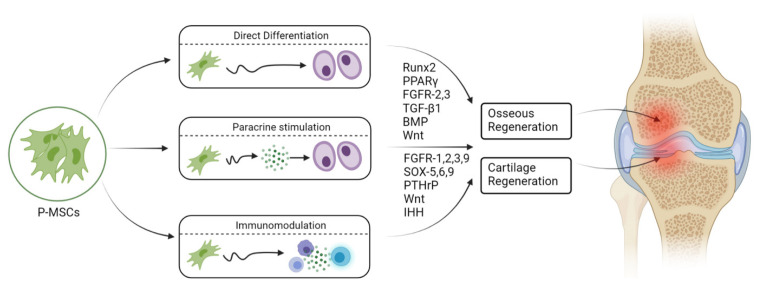
Osteogenic and chondrogenic differentiation of periosteal mesenchymal stem cells. Created with BioRender.com.

**Figure 3:**
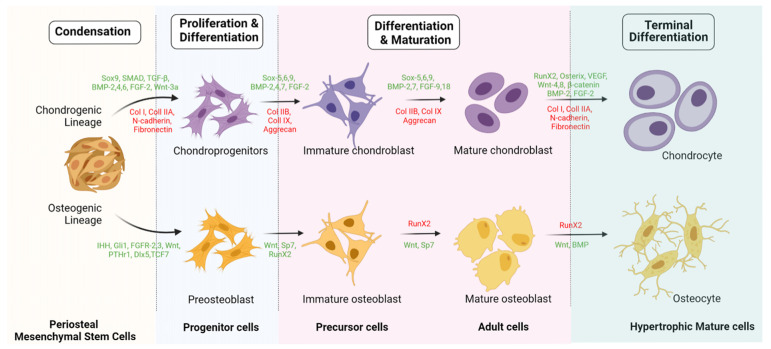
Intracellular signaling of osteogenic and chondrogenic differentiation of periosteal mesenchymal stem cells. Created with BioRender.com.

The lineage differentiation potential of MSCs is mainly governed by PPARγ and Runx2 genes. Runx2 is the key regulator for pro-osteogenesis, whereas PPARγ is responsible for anti-osteoblastogenic effects [61]. Runx-2 and -3 are responsible for osteogenesis and chondrogenesis of MSCs. Along with Runx-2, TGF-β1, BMP, Wnt, Hedgehog (HH), and (Nel)-like protein type 1 (NELL-1) activate and regulate osteogenic responses, as shown in Figure 2 and Figure 3 [62,63].

A dysfunctional β-catenin pathway results in impaired osteoblastic maturation and mineralization [64]. The Wnt/β-catenin signaling pathway leads to downregulated osteoclastogenesis and bone resorption [65]. Antagonists of Wnt signaling molecules such as anti-sclerostin (SOST) and anti-dickkopf-1 enhance MSC-dependent osteogenesis and upregulate bone mineral density [66,67].

Macrophage lineage progenitors recruit P-MSCs for cortical osteogenesis. Colony-stimulating factor knockout results in dysregulated recruitment of macrophage progenitors, leading to impaired periosteal osteogenesis [33]. TRAP^+^ macrophages induce the expression of periostin and P-MSC recruitment to the periosteal surface through platelet-derived growth factor-BB (PDGF-BB). In the mice model, cortical bone osteogenesis was exhibited by P-MSC-derived Nestin^+^ and LepR^+^ CD45^−^ Ter119^−^ CD31^−^ cells. Hence, macrophage lineage progenitors play a significant role in periosteal homeostasis [33]. The upregulation of periostin by PDGF-BB was induced by PDGFR-β, PI3K, AKT, and CREB phosphorylation [15,68]. Periostin, along with autophosphorylated PDGFR-β, regulates and maintains periosteal osteogenesis [15].

Intraosseous 5 μg BMP-2 injection in a mouse model of osteogenesis imperfecta resulted in periosteal ossification and enhanced biomechanical strength and thickness of cortical bone [69]. Endosteal and periosteal osteogenesis was ascertained in chronic ovariectomized female cynomolgus monkeys with an intraosseous injection of recombinant BMP-2 loaded into calcium phosphate matrix into the femoral neck [70]. In a mouse model of spondyloarthropathy, IL-17A deficiency attenuated inflammatory cytokines and bony erosions and promoted periosteal osteogenesis [71]. IL-17A inhibited osteoblastic differentiation in inflammatory periosteum by inducing Wnt antagonist secreted frizzled-related protein (sFRP)-1 and suppressing sFRP-3 expression [72,73,74]. Beta tricalcium phosphate block induced periosteal osteogenesis and acted as a space maker in the soft tissue defect [75].

## 6. Engineered Osteogenesis by P-MSCs

The key challenge in tissue engineering is the formation of new blood vessels (neovasculogenesis) at the transplanted site. Van Gastel et al. observed increased proangiogenic potential when murine P-MSCs were cotransplanted with a collagen calcium phosphate scaffold and endothelial cells in vivo [76]. As a result, they exhibited pericyte-like cells which induced hematopoietic stroma with neovasculogenesis [76]. Zheng et al. observed enhanced osteogenesis in the form of increased expression of osteocalcin, osteonectin, and type 1 collagen in 3D culture when P-MSCs were loaded onto a poly(lactic-*co*-glycolic acid) scaffold with allogenic serum [77]. Enzymatically harvested and culture-expanded periosteal cells from human rib periosteum exhibited in vitro osteogenesis and chondrogenesis [78].

Cell-specific COX-2 gene deletion leads to inhibition of BMP-2-mediated differentiation lineages of osteogenesis, chondrogenesis, and vasculogenesis in P-MSCs. Gene profiling exhibited the downregulation of genes responsible for osteogenesis and chondrogenesis such as SOX-9, MMP-9, Osx, Runx-2, and RANKL. COX-2-deficient cells displayed downregulation of HIF-1, PI3K-AKT, and Wnt pathways [79]. Sostdc-1 gene expression was observed in the periosteum, which serves as an important factor in fracture remodeling. Dysregulation of the Sostdc-1 gene resulted in acceleration of fracture healing by promoting the expansion of P-MSCs [80]. They enhanced and maintained quiescent MSCs of the periosteum [80,81].

Administration of a prostaglandin E1 receptor antagonist in wild mice enhanced the production of CFUs of periosteal cells, osteoblastic CFUs, and osteoblastic differentiation of P-MSCs. This enhanced the callus formation in fracture by 10 days [82]. Bravo et al. emphasized that inhibition of plasminogen activator by epsilon aminocaproic acid (EACA)-treated P-MSCs promoted osteogenesis during hard callus formation in appendicular skeleton fractures [83]. The evaluation of callus treated with EACA exhibited increased Wnt and BMP signaling and reduced TGF-β-signaling. EACA treatment enabled a robust switching from chondrogenesis to enhanced osteogenesis, which changed the fate of osteoprogenitor cells of the periosteum [83]. Fibrin admixed with P-MSCs enhanced the proliferation of osteogenic precursors. Tagging of tranexamic acid along with fibrin constructs maintained the integrity of the osteogenic differentiation of P-MSCs [84].

In hypoxic conditions, the enhanced level of H3K27me3 in the promoter region of BMP-2, in combination with decreased KDM6B activity, resulted in inhibition of osteogenic progenitors of P-MSCs [85]. Dysregulation of the Lin28a gene, an RNA-binding protein, resulted in the suppression of osteogenesis and mitochondrial activity of P-MSCs [86]. Jumonji domain-containing 3 (Jmjd3), a histone demethylase, induces osteoblastic differentiation and regulates the expressions of BSP and OCN via transcription factors Runx2 and osterix [87]. Jmjd3 maintains the stemness of MSCs in the periosteum [87,88]. Jagged-1, a Notch ligand, downregulates the osteoprogenitor pool and periosteal expansion along with homotypic notch signals and upregulates trabecular bone formation [89].

Enhanced osteogenesis was observed when MSCs were seeded with an ECM sheet containing calcium phosphate nanoparticles and growth factors such as ANG-1, TGF-β1, bFGF, and VEGF. Such engineered MSC populations act as a biomimetic periosteum that can be used in critical bone defects [90]. Integration of vascular bone graft by an engineered periosteal sheet and β-TCP scaffold mimicked the cellular configuration of the periosteum and, hence, enhanced neo-osteogenesis and neovasculogenesis in bone tissue regeneration [91]. Injectable periosteal ECM activated macrophage (M2) polarization and enhances MSC differentiation into osteogenic cells in calvaria defects in rats [92].

## 7. Intracellular Signaling in Chondrogenic Differentiation

Various researchers have demonstrated the chondrogenic differentiation of MSCs in vitro with the addition of external biological micro-molecules such as growth factors, bone morphogenetic proteins (BMPs), Hedgehog, SOX signaling molecules, transcription factors, matrix proteins, and Wnt glycoproteins.

Co-expression of SOX-9 with SOX-5 and -6 activates chondrogenic differentiation. Knockout of SOX-9 results in chondrocyte hypertrophy. Repression of chondrocyte hypertrophy by SOX-9 is due to expression of VEGF antagonist and COL10A1 in hypertrophied chondrocyte and inhibition of Runx-2 activation.

Wnt (Wnt5a and Wnt5b) signal regulation of chondrogenesis results in controlled longitudinal growth of long bones by regulating cyclin D1, p130, and chondrocyte-specific COL2A1 expression. Yang et al. stated that Wnt5a and Wnt5b signals control the proliferation of chondrocytes in different zones of cartilage tissues [93]. Wnt5a upregulates and Wnt11 downregulates chondrogenesis via the exposure of chondrocytes to IL-1β. Such transcription results in the inhibition of collagen type 2 expression in chondrocytes [94]. Through BMP-dependent signaling, Twist-1, transcription suppressor gene, inhibits chondrogenesis and chondrocyte gene expression [95].

Fibroblast growth factor (FGF) is an important factor in endochondral and intramembranous bone development, where it regulates proliferation and hypertrophy of chondrocytes. FGF-R1 and -R2 exhibit a chondrogenesis lineage, whereas FGF-R1 is expressed in higher quantities in hypertrophied chondrocytes and FGF-R2 downregulates proliferating chondrocytes. FGF-R3 upregulates chondrocyte proliferation and downregulates hypertrophic chondrocytes. FGF-R9 promotes chondrocyte proliferation and hypertrophy in early stages, whereas it regulates neoangiogenesis in later stages. Indian Hedgehog (IHH) gene and PTHrP signaling work simultaneously in chondrogenesis, as shown in Figure 2 and Figure 3. The IHH gene promotes chondrocyte proliferation and controls PTHrP expression. Hyperexpression of PTHrP exhibits delayed chondrocyte differentiation.

Sustained activation of NF-κB releases nitric oxide, which induces catabolism of cartilage and results in cartilage degeneration [96]. Changes in the oxygen tension have profound effects on chondrocyte differentiation, gene expression, and morphology, along with their production and response to cytokines in the environment. Although hypoxia is a necessary factor in their development, low oxygen tension levels inhibit glycolysis, induce negative Pasteur effects, and inhibit matrix production due to the alteration in the levels of reactive oxygen species (ROS) [97]. Cartilage oligomeric matrix protein (COMP) inhibits BMP-2-induced MSC-bound osteogenesis as evidenced by the evaluation of COL1A1, Runx-2, OPN, and bone Gla protein, while it enhances BMP-2-induced MSC bound chondrogenesis as evidenced by the evaluation of COL2A1, SOX-9, and aggrecan [98].

Guerit et al. exhibited that the upregulation of FOXO3A and downregulation of miR-29a are essential for MSC differentiation into chondrocytes and in vivo osteochondral formation [99]. Upregulation of Wnt signaling promotes the transcription of miR-140-5p in MSC-bound chondrogenic lineage and inhibits cartilage degradation and inflammation [100]. Enhanced expression of miR-145 negatively regulates chondrogenesis by decreasing mRNA levels of chondrogenic markers such as COL2A1, aggrecan, COMP, COL9A2, and COL11A1 in murine MSCs induced by TGF-β3 [101]. miR-194 downregulation enhances AD-MSC-mediated chondrogenesis by targeting the Sox-5 gene [102]. miR-199a, a BMP-2 encoded miRNA, enhances MSC-mediated chondrogenesis by targeting the Smad-1 gene [70,103].

Enhancement of early chondrogenesis and inhibition of hypertrophic chondrocyte differentiation is promoted by low oxygen tension. Portron et al. emphasized that hypoxia prevents calcification of MSC-bound chondrogenesis [104]. Under chondrogenic mediators (hypoxia) influences, MSCs were cultured in a fibrin glue scaffold to observe the proliferation of rounded chondrocyte-like cells with the chondral phenotype; it was concluded that hypoxia is the better stimulant for chondrogenesis [105].

In the collagen-induced arthritis model, small hyaluronic acid fragment activity inhibition and A2A adenosine receptor pathway stimulation limited apoptosis and reduced cartilage damage [106]. MSCs complexed with nonviral vector polyethyleneimine (PEI), mediated by SOX-5, -6, and -9 and loaded onto poly(lactic-*co*-glycolic acid) (PGLA) nanoparticles, resulted in enhanced chondrogenesis of human MSCs in vitro in culture media [107]. Codelivery of chondrogenic mediators such as SOX-9 and anti-Cbfa-1 siRNA loaded onto PGLA nanoparticles exhibited MSC-bound enhanced chondrogenesis [108].

## 8. Chondrogenicity of P-MSCs

Ito et al. studied the chondrocyte precursors in the periosteum, and their observations were as follows: (a) the cambium layer of the periosteum caters to chondrocyte precursors, (b) appositional neochondrogenesis displaces the fibrous layer away from already formed cartilaginous tissue, and (c) chondrogenesis commences from the juxtaosseous to juxtafibrous region of the cambium layer [14].

Periosteal progenitors possess chondrogenic potential in the presence of chondrogenic-dependent transcriptional factors and signaling pathways. In the presence of TGF-β3, periosteal progenitors differentiate into chondrocytes along with atelocollagen, which was further evaluated by immunohistochemical staining for type 2 collagen [109].

Downregulation of neural cell adhesion molecule (NCAM) expression during periosteal cell commitment during secondary chondrogenesis provides an alternate pathway for periosteal chondrogenesis [110]. O’Driscoll et al. exhibited that the chondrogenic potential for periosteal MSCs declined with aging in a 12 month old rabbit model by 87% [111]. P-MSCs from the rat model exhibited chondrogenesis due to the induction of BMP-2, whereas terminal chondrocyte differentiation was modulated by TGF-β1 [112].

Higher expression of FGF-16 and -18 was observed in periosteal chondrogenesis of callus formation [59]. Downregulation of Notch-2 expression was observed during chondrogenesis of P-MSCs [60]. Subperiosteal injections of TGF-β1 either alone or in combination with IGF-1 rejuvenated aged periosteum in the rabbit model by increasing the cellular count in the cambium layer and via in vitro chondrogenesis [113].

Shreds of evidence prove that Wnt signaling inhibitors enhance early chondrogenesis, which was demonstrated by a glycosaminoglycan assay, as well as SOX-9, and COL2A1 gene expression. In long-term culture and later in chondrogenesis, Wnt signaling does not play any role in the cartilage tissue engineering of MSCs [114].

## 9. Engineered Chondrogenesis of P-MSCs

Various studies have emphasized that the periosteum predominantly gives rise to chondrocytes [115,116]. Brittberg et al. demonstrated that the periosteum possessed a higher degree of clonogenicity when chondrocytes were cocultured with periosteal cells and agarose [116]. Such cocultured chondrocytes expressed higher levels of IL-6 and -8, TGF-β3, and GM-CSF. They concluded that TGF-β3 can promote periosteal chondrogenesis. P-MSC chondrogenesis and MSC differentiation were observed when TGF-β3 was injected subperiosteally [117,118,119]. Periosteal cells from adults and the elderly population retain proliferation and multilineage differentiation capacity. Subperiosteal injection of TGF-β1 and IGF-1 increased the cellular count and phenotypic stability in the cambium layer of periosteum in aged rabbits, as well as in vitro cartilage regeneration [117]. In vitro cultured rabbit periosteal explants exhibited chondrogenic potential when exposed to TGF-β1 [120]. Cultured chondrocytes showed increased expression of type 2 collagen.

Digoxin and ATP increase collagen content in neocartilage by around 110%. The tensile strength of newly formed cartilage was increased by digoxin (280%) and ATP (180%). Digoxin and ATP increased Ca^2+^ oscillations in monolayer cultured chondrocytes. Hence, calcium modulators enhance neochondrogenesis [121]. Evidence has shown that the supplementation of ECM materials such as COMP and aggrecan in a biogel formulation (low-melting-point agarose) mimicked the composition of the cartilage tissue when injected in between bone and the periosteum of the superomedial aspect of rabbit tibia. Regenerated subperiosteal chondrogenic tissue was analyzed for glycosaminoglycan and DNA content, as well as ALP activity. ALP activity was grossly decreased with an increase in GAG and DNA content in regenerated cartilage. This model created a suppressive environment for the chondrocyte hypertrophy niche with chondrogenic progenitors for cartilage tissue engineering in in vitro bioreactors [122]. Hence, this milieu provides a broad-based platform for cartilage grafting in cartilage defects.

In the presence of TGF-β3 in in vitro cultures of periosteal cells, aggrecan induces the chondrogenic potential of periosteal cells and regenerates in vitro bioreactor cartilaginous tissue [123,124]. Such neocartilaginous tissues were able to exhibit GAG with increased TGF-β3 signals [125]. These models suppressed chondrocyte hypertrophy via NKX3-2/Bapx-1 expression [126]. Addition of anti-osteogenic reagents such as fulvestrant and IL-1β to the vascularized periosteum enhanced cartilage regeneration by downregulating osteogenesis [127]. P-MSCs mediated chondrogenesis when administered a cocktail of thermoreversible gelation polymer (TGP) (poly(*N*-isopropyl acrylamide)), polyethylene oxide, and TGF-β3. The combination of P-MSCs and TGP injection provides a better biocompatible complex for injured cartilage as a minimally invasive procedure [128]. The enhanced expression of TGF-β1 and COL1A1 genes in periosteal paracrine cocultures and the enhanced release of TGF-β1 induced chondrogenesis in an in vitro model of P-MSC-based autologous chondrocyte implantation [129].

In a rib fracture mouse model, Li et al. demonstrated the early stages of chondrogenesis and osteogenesis of periosteal cells. They demonstrated the potential of P-MSCs from osteoblastic differentiation into a chondrogenic lineage and the involvement in osteocytes in osteochondral regeneration by P-MSCs [130]. Downregulation of α1 integrin and upregulation of α3 integrin by threefold, of α5 integrin by threefold, and of β1 by fourfold were observed during extracellular matrix synthesis by periosteal cells. These markers were confirmed in periosteal chondrocytes by immunohistochemistry methods [131]. Injection of TGF-β1 using poly-ε-caprolactone nanofiber scaffolds seeded in vivo with P-MSCs resulted in chondrogenesis in 6 month old rabbits. The chondrocyte yield from P-MSCs was significantly increased by subperiosteal TGF-β1 injections [132].

## 10. Future Perspectives

Having explored the comparative osteogenic, chondrogenic, and adipogenic potential of P-MSCs in vitro in comparison to other potential sources such as bone marrow MSCs [133], clinical trials on similar grounds are needed to validate the results of the in vitro studies. Apart from periostin, other potential key mediators in the cellular molecular mechanism involved in the mechanosensing and intrinsic repair capabilities of P-MSCs need identification to probe the molecular mechanisms underlying their function. P-MSCs were identified to form an osteoid complex via an intramembranous route, which provides a cellular divergence between the developmental pathways of bone formation [134].

The concept of tissue-engineered periosteum (TEP) is being evaluated for scenarios where the native periosteum is damaged with very few P-MSCs to orchestrate the repair mechanisms. Currently, few studies have been done to characterize the mechanical properties of TEP, and they are being used successfully in oral applications rather than a high-demand site such as long bones of the body [4]. This tissue engineering approach with 3D scaffolds as a periosteal mimetic for cellular attachment, migration, and proliferation needs further validation for practical applicability toward routine clinical use. Furthermore, the identification of factors that regulate stem-cell quiescence and activation is needed. Identification markers of P-MSCs from the resident cambium layer are also needed.

## 11. Conclusions

The role of P-MSCs in the anabolic pathways of osteochondral tissue repair was highlighted. With a deeper understanding of the mechanosensing and key mediators driving the differentiation pathways, TEP could be used as a substitute in post-traumatic and degenerative conditions of the bone. P-MSCs possess both osteogenic and chondrogenic potential, which is of high therapeutic value. However, clinical validation studies on their utility in various scenarios need to be undertaken to mark their comparative benefit across the various common sources of MSCs.

## Data Availability

Data is contained within the article.
